# A case of “ainhum”: a rare clinical image

**DOI:** 10.11604/pamj.2023.44.129.39066

**Published:** 2023-03-15

**Authors:** Madasu Sai Vikas

**Affiliations:** 1 Department of General Surgery, Jawaharlal Nehru Medical College, Acharya Vinoba Bhave Rural Hospital, Datta Meghe Institute of Higher Education and Research, Sawangi, Wardha, Maharashtra, India

**Keywords:** Ainhum, dactylolysis spontanea, amputation

## Image in medicine

The word “ainhum” comes from an African word that means “to saw or cut”. True ainhum, also known as dactylolysis spontanea, is a disorder that affects soft tissue or digits with constricting rings and typically manifests in the fifth toe. We describe a rare instance of genuine ainhum that only affected the left fifth toe. Few cases have ever been reported globally, and this one is extremely rare. South Africa and South America have been found to have the highest incidence of ainhum. In India, it is seldom ever recorded. Ainhum when diagnosed and treated in the early stages can be prevented from progressing to mutilating deformities. In the case presented here, a 60-year-old woman complained of pain and swelling on her left fifth toe, which was free of any ulceration or discharge. The patient described a minor injury to the left fifth toe two months prior to seeking our help, which was followed by altered sensation before the situation eventually worsened to what it is now, with no signs of gangrene or other skin abnormalities. Every peripheral pulse was felt. He lacked any localized lymphadenopathy. Upon closer inspection, it was discovered that the left fifth toe's proximal interphalangeal joint level had a band-like soft tissue constriction. An X-ray of the left foot revealed a constricting band that was so deep that it separated the distal segment almost to the point of auto-amputation in the middle of the proximal phalanx.

**Figure 1:**
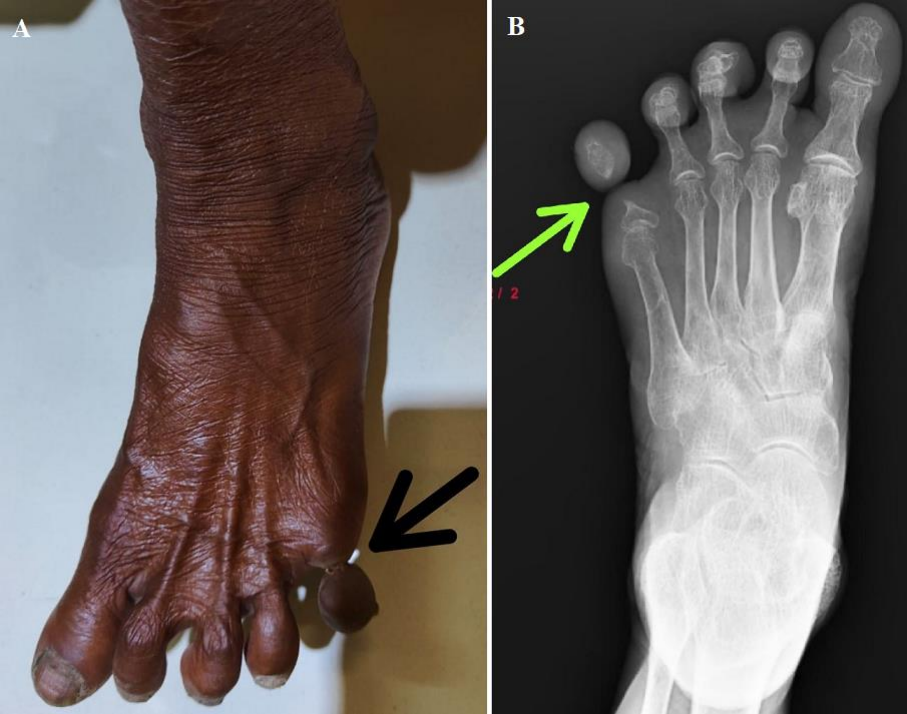
A) “ainhum”; B) X-ray of left foot

